# Drug Synergy Drives Conserved Pathways to Increase Fission Yeast Lifespan

**DOI:** 10.1371/journal.pone.0121877

**Published:** 2015-03-18

**Authors:** Xinhe Huang, Markos Leggas, Robert C. Dickson

**Affiliations:** 1 Department of Molecular and Cellular Biochemistry and the Lucille Markey Cancer Center, University of Kentucky College of Medicine, Lexington, Kentucky, United States of America; 2 Department of Pharmaceutical Sciences and the Lucille Markey Cancer Center, College of Pharmacy, University of Kentucky, Lexington, Kentucky, United States of America; University of Cambridge, UNITED KINGDOM

## Abstract

Aging occurs over time with gradual and progressive loss of physiological function. Strategies to reduce the rate of functional loss and mitigate the subsequent onset of deadly age-related diseases are being sought. We demonstrated previously that a combination of rapamycin and myriocin reduces age-related functional loss in the Baker’s yeast *Saccharomyces cerevisiae* and produces a synergistic increase in lifespan. Here we show that the same drug combination also produces a synergistic increase in the lifespan of the fission yeast *Schizosaccharomyces pombe* and does so by controlling signal transduction pathways conserved across a wide evolutionary time span ranging from yeasts to mammals. Pathways include the target of rapamycin complex 1 (TORC1) protein kinase, the protein kinase A (PKA) and a stress response pathway, which in fission yeasts contains the Sty1 protein kinase, an ortholog of the mammalian p38 MAP kinase, a type of Stress Activated Protein Kinase (SAPK). These results along with previous studies in *S*. *cerevisiae* support the premise that the combination of rapamycin and myriocin enhances lifespan by regulating signaling pathways that couple nutrient and environmental conditions to cellular processes that fine-tune growth and stress protection in ways that foster long term survival. The molecular mechanisms for fine-tuning are probably species-specific, but since they are driven by conserved nutrient and stress sensing pathways, the drug combination may enhance survival in other organisms.

## Introduction

Most organisms show signs of aging, characterized by the gradual but progressive loss of physiological functions over time. As humans enter their fifties and sixties the consequences of such functional losses manifest as increased incidence of cancer, type 2 diabetes, neurodegeneration, cardiovascular disease and immune dysfunction, the age-related diseases. Current research seeks to reduce the incidence and severity of these diseases to improve health in the elderly. One promising avenue of research centers on the target of rapamycin complex 1 (TORC1) protein kinase [1–3]. TORC1 is a central regulator of aging and longevity, since down-regulating its activity by treatment with the natural product rapamycin or related synthetic compounds, reduces signs of aging and extends lifespan in model organisms ranging from yeast to mice [4–8]. These seminal results have spawned an array of TOR-related research including work from our laboratory which identified a strategy that produces a synergistic increase in the chronological lifespan (CLS) of the Baker’s yeast *Saccharomyces cerevisiae* [9]. This being the first example of a true synergistic increase in lifespan produced by drugs. Our strategy uses a low dose of rapamycin to lower (not inhibit) TORC1 activity and another natural product, myriocin, to lower (not inhibit) the activity of serine palmitoyltransferase (SPT), the first enzyme in the sphingolipid biosynthesis pathway. The conserved nature of TORC1 and SPT in eukaryotes suggested that the combination drug treatment (ComboDT) might enhance lifespan in other eukaryotes. Here we verify this prediction by using the fission yeast *Schizosaccharomyces pombe*, a distant and divergent relative of budding yeast.

Most eukaryotes have two TOR complexes with TORC1 primarily responsible for sensing nutrients, stresses, growth factors, and energy status and coupling these to growth and survival including lifespan whereas TORC2 responds to growth factors and controls cytoskeletal functions but has no known role in lifespan [1, 2, 10]. Rapamycin primarily inhibits TORC1 and at high doses it inhibits growth. However, it does effect TORC2 in some organisms such as mammals [1]. Rapamycin does not inhibit growth in fission yeasts probably because of incomplete inhibition of TORC1 activity, but combining rapamycin with caffeine completely inhibits enzyme activity and growth [11]. Despite incomplete inhibition of TORC1, rapamycin treatment extends CLS in fission yeast as does treatment with caffeine [12], similar to the effect of caffeine on budding yeast [13]. In contrast to ComboDT, rapamycin and caffeine in combination are no more effective in increasing fission yeast lifespan CLS than are the single drugs.

Sphingolipids have many functions in eukaryotes including roles in age-related human diseases [14–17]. In *S*. *cerevisiae* they can be modulated to increase both CLS, a measure of survival in non-dividing cells, and replicative lifespan (RLS), a measure of how many times a cell can divide [reviewed in [18]]. For example, treatment with a low dose of myriocin to reduce SPT activity increases CLS in *S*. *cerevisiae* [19] and treatment with even lower doses of myriocin in combination with low doses of rapamycin produce a synergistic increase in CLS [9]. It is not clear how such modulation of sphingolipids increases CLS or RLS, but it is likely to be complex and involve changes in sphingolipids that act as second messengers as well as effects on cellular processes connected to membranes including membrane trafficking and the functionality of membrane-bound proteins.

What we do know is that myriocin and ComboDT increase budding yeast CLS by controlling conserved nutrient sensing and stress response signaling pathways including, besides TORC1, the protein kinase A (PKA) and AMP kinase (AMPK) pathways [9, 19]. In addition, we know that myriocin [20] and ComboDT (unpublished data) modulate gene expression and cellular processes (GO terms) in ways that are similar to what is found with rapamycin treatment and dietary or calorie restriction (CR), the gold standards for slowing aging and enhancing lifespan.

With this information along with current knowledge of *S*. *pombe* aging and lifespan as background, we examined the effect of ComboDT on fission yeast and show that the drugs enhance CLS and do so in a genuinely synergistic fashion. As predicted, the drug combination enhances CLS by using signaling pathways with known roles in aging and lifespan. These include the TORC1 pathway [12] and its downstream protein kinase substrates [21–24], the nutrient-sensing PKA pathway [21] and the Sty1 stress response pathway, [24, 25]. Sty1 is the fission yeast ortholog of the Hog1 kinase of *S*. *cerevisiae*, which controls responses to osmotic stress [reviewed in [26, 27]]. Sty1 is also an ortholog of the mammalian p38 MAP kinase, a Stress Activated Protein Kinase (SAPK) with strategic roles in inflammation and stress responses and more recently discovered roles in cell cycle regulation, proliferation and senescence [reviewed in [28]].

One of the TORC1 substrates with well documented roles in aging and lifespan is the conserved S6 kinase, prime examples being Sch9 in *S*. *cerevisiae* [29, 30] and S6K1 and S6K2 in mammals [31, 32]. The S6 kinase orthologs in fission yeasts appear to be Psk1, Sck1 and Sck2 [33]. We find that all of these kinases are involved in CLS enhancement by ComboDT. Together, our results support the premise that ComboDT enhances lifespan by controlling signaling pathways which regulate conserved cellular processes in ways that have evolved to meet species-specific needs for long term survival.

## Results

For culture medium we used yeast extract plus supplements (YES) in our studies as it is used most often for assaying CLS in fission yeast [12, 22–24]. While 3% glucose is often used for culturing fission yeasts, we used 2% because it reduces adaptive regrowth in fission yeasts [21] and is used extensively in studies of budding yeast lifespan [34]. Preliminary dose response studies showed that neither myriocin nor rapamycin impaired the cell density achieved at 48 hrs of growth (S1A and S1B Fig.), the time at which CLS assays were initiated. Likewise, the concentration of drugs used in ComboDT (150 nM or 60 ng/ml myriocin and 50 nM or 46 ng/ml rapamycin) did not interfere with cell density at 48 hrs (S2 Fig.) and, in fact, cells treated with myriocin or ComboDT grew to a slightly higher density than without drug treatment, similar to what we have observed in budding yeasts [9, 19]

### Synergistic increase in lifespan

**Fig 1:**
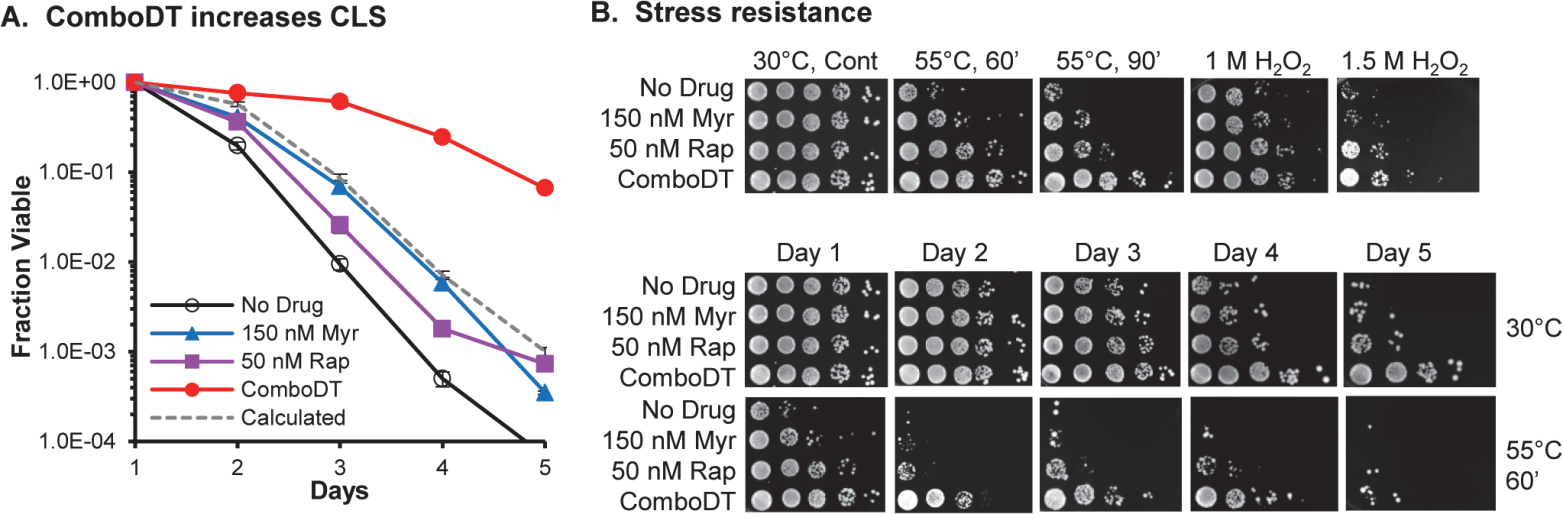
ComboDT increases lifespan and stress resistance. (A) Viability for 972 h^-^ cells is shown for values normalized to CLS day 1 (48 hrs of incubation at 30°). Data are the mean ± SEM of viable cells in triplicate cultures in these and all other lifespan data sets. The dashed line represents the small enhancement in viability for the single drugs values added to the WT value at each time point. Values for statistical significance are given in S2 Table. (B) The spot-dilution (10-fold from left to right) assays in the upper panel show the response of cells on CLS day 1 to heat or hydrogen peroxide stress while the lower panel shows heat stress resistance at each day of the CLS assay.

To determine if ComboDT can produce a synergistic increase in *S*. *pombe* CLS, we searched for low concentrations of each drug showing little or no effect on lifespan but that produced a large and potentially synergistic increase in lifespan when used in combination. An example of the effect of one such combination on cell survival is shown in Fig. 1A. To assess whether the increase in CLS produced by this combination of drugs is synergistic, we performed survival experiments similar in design to those used to develop drug combinations that produce a synergistic decrease in the viability of cancer cells. Survival was measured after growing cells to stationary phase (48 hrs, CLS day 1) and then again after 32 hrs of incubation, since at this time viability in untreated cells had decreased by 90% allowing enhancement caused by the ComboDT to be evaluated over a large concentration range (Table 1, S3A and S3B Fig., for dose response plots).

**Table 1:** Myriocin and rapamycin generate a synergistic increase in lifespan.

Combination Treatment Concentration (nM)		Single Treatment Concentrations (nM) †			Synergy is defined as:			Molar ratio
Rap	Myr	Rap	Myr	Survival (fold over control)¶	CI < 1Chou	α > 0 Greco	β_4_ > 0 Plummer	Myr/Rap
50	15	251	90.3	2.33	0.366	28.4	3.485	0.3
100	30	332	102	2.67	0.597	5.51	1.351	0.3
200	60	2180	130	3.44	0.555	4.41	2.161	0.3
400	120	2180	196	5	0.795	0.62	0.612	0.3
50	50	2180	203	5.11	0.27	43.3	9.704	1
100	100	2180	375	7.44	0.312	13.9	6.219	1
200	200	2180	645	8.89	0.402	3.96	3.548	1
400	400	2180	320	6.89	1.434 §	-0.506	-0.906	1
**50‡**	**150**	**2180**	**966**	**9.56**	**0.178**	**35.5**	**13.77**	**3**
100	300	2180	450	8	0.713	2.11	1.639	3
200	600	2180	612	8.78	1.072	-0.156	-0.242	3
400	1200	2180	823	9.33	1.64	-0.399	-1.238	3

† Values represent the concentration of individual drugs necessary to produce the same degree of survival as the combination treatment and were calculated from dose response curves.

¶ Survival represents the fraction of cells viable after 48 hrs of incubation (CLS Day1) divided by the survival after a further 32 hrs of incubation.

‡ The drug combination (150 nM myriocin + 50 nM rapamycin = 60 ng/ml myriocin + 46 ng/ml rapamycin) shown in this row was used to generate the lifespan data shown in Fig. 1A and other experiments labeled with the term ComboDT.

§ Underlined values represent drug combinations in the corresponding row that give additive or antagonistic effects.

The effect of myriocin and rapamycin alone on survival was assessed to obtain the median effective concentrations (EC50) for each drug. The EC50 of rapamycin was 221 nM and for myriocin it was 231 nM. Non-linear regression was used to fit the survival verses concentration data (S3A and S3B Fig.) and the resulting curves were used to determine the concentrations that would be required for each drug alone to elicit the same survival effect as observed with combinations of rapamycin and myriocin in different molar ratios (Table 1). These data were used to calculate the combination index (CI) according to Chou and Talalay [35]. To determine how robust the CI results were, we calculated the alpha and beta4 parameters used to determine deviation from additivity in the more restrictive models of Greco and Plummer, respectively [36, 37]. All mathematical models predict synergy (Table 1). Surprisingly, the molar ratio of the two drugs has no effect on synergy whereas their concentration does. The effect approaches additivity as the rapamycin concentration approaches 400 nM and myriocin approaches 100 nM. Conversely, concentrations of 100 nM rapamycin and 300 nM myriocin approach additivity. Below those concentrations, for both drugs, there is strong synergy and above those concentrations, for both drugs, there is antagonism (Table 1, underlined values).

Improved stress resistance is associated with increased lifespan in many organisms [38, 39]. This correlation was first observed in fission yeast carrying mutations in nutrient signaling pathways [21] and recently in cells treated with rapamycin or caffeine or both to inhibit TORC1 activity [12, 40]. To determine if ComboDT increases stress resistance we examined resistance to oxidative (hydrogen peroxide) or heat stress on CLS day 1. ComboDT cells were more than ten-fold resistant to heat stress than either drug alone (Fig. 1B, top panel) and such resistance to heat stress persisted from day 1 through day 5 (Fig. 1B, bottom set of panels—compare the 30°C control samples to the 55°C heat stressed samples). ComboDT slightly increases (∼2-fold) resistance to hydrogen peroxide stress compared to rapamycin alone and increases resistance more than 10-fold compared to myriocin alone (Fig. 1B, top panel).

### TORC1 Signaling and Stress Resistance

**Fig 2:**
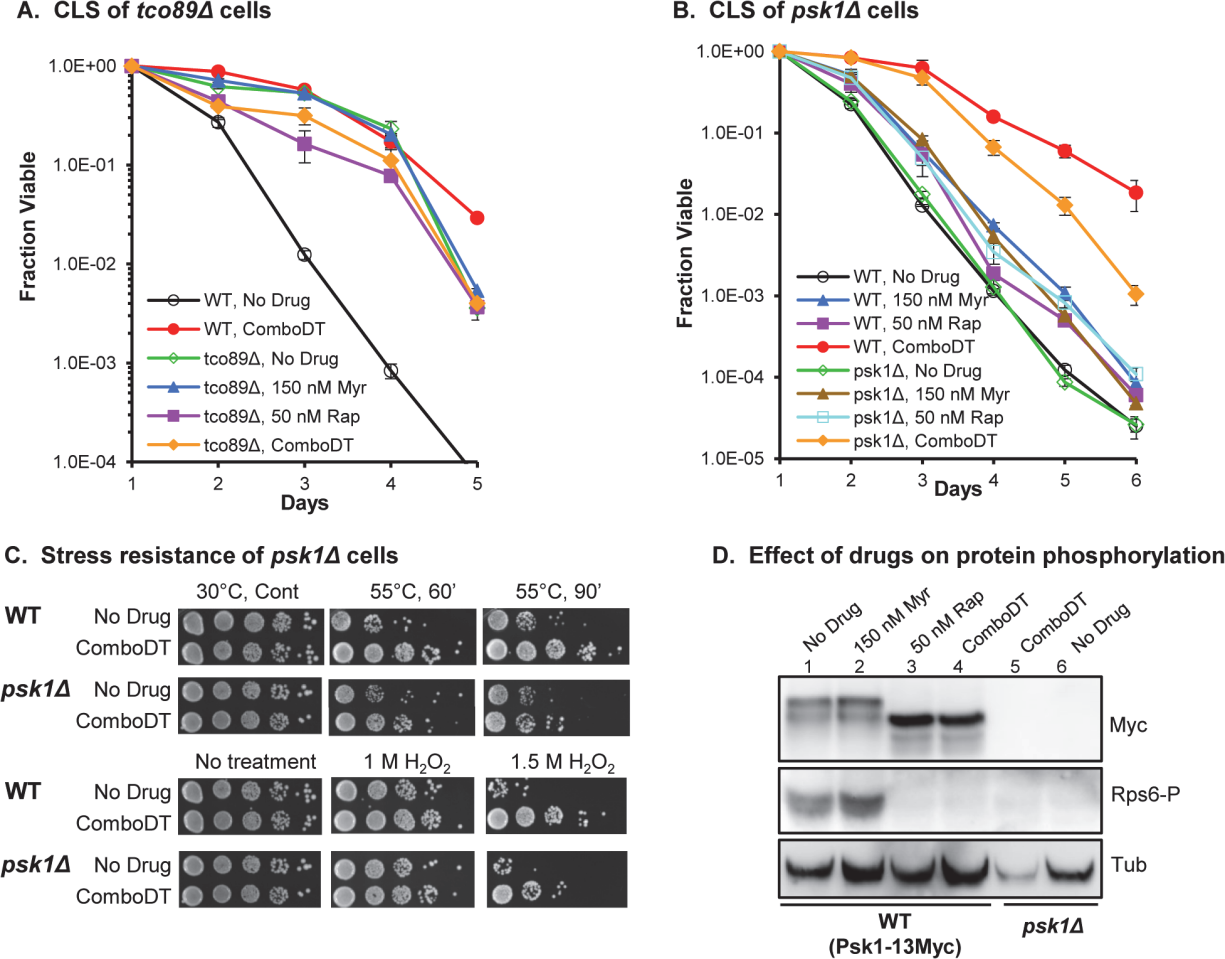
ComboDT requires the TORC1-Psk1 pathway to extend lifespan. (A) Effect of deleting the *tco89* subunit of TORC1 on CLS in cells +/- drugs. (B) Effect of deleting the *psk1* gene, encoding an S6 kinase substrate of TORC1, on CLS. (C) Effect of deleting the *psk1* gene on heat and hydrogen peroxide stress resistance. (D) The effect of drugs on TORC1 activity was determined by measuring TORC1-mediated phosphorylation of Myc-tagged Psk1 and the Psk1 substrate protein Rps6. Tubulin (Tub) served as a loading control for samples shown in each lane of the bottom immunoblot while the two upper blots show the concentration of Psk1–13Myc (Myc) or phosphorylated Rps6 (Rps6-P).

Because TORC1 controls aging and longevity in many organisms [1, 3, 41] including regulation of CLS in fission yeast [12], we determined if ComboDT decreases TORC1 activity as a way to extend lifespan. Tco89 is a nonessential core subunit of TORC1 and deletion of the *tco89* gene, which decreases TORC1 activity, enhances CLS in fission yeast similar to what is seen in WT cells treated with rapamycin [12]. Moreover, treatment of *tco89Δ* cells with rapamycin does not further increase CLS, indicating that TORC1 plays a key role in lifespan. We also find that *tco89Δ* cells live longer than WT cells and that neither ComboDT nor the single drugs further increase CLS in a statistically significant way (Fig. 2A), consistent with ComboDT enhancing CLS by decreasing TORC1 activity. One unique feature of ComboDT is that it promotes survival of WT cells at day 5 better than does deletion of *tco89*, suggesting, and as we show below, that ComboDT uses both TORC1-dependent and-independent mechanisms to enhance CLS. Unexpectedly, when rapamycin is used alone at the low concentration contained in ComboDT there is a small, but statistically significant reduction in the CLS of *tco89Δ* cells. This effect of rapamycin was not seen previously [12] and may be due to one or more small differences in experimental protocols including glucose concentration (Rallis et al. vs. our work: 3% vs. 2%), aeration/oxidative stress (shaker rpm 130 vs. 220), incubation temperature (32°C vs. 30°C) and initial cell density (A600nm of 0.15 vs. 0.005).

To explore further the influence of ComboDT on the TORC1 pathway, we examined TORC1 substrates. The S6 kinases, exemplified by the Sch9 kinase in *S*. *cerevisiae* [29, 30] and S6K1 and S6K2 kinases in mammals [32], are one of the best characterized TORC1 substrates. There has been debate about which *S*. *pombe* kinases are actual S6K orthologs, but at this time they appear to be Psk1, Sck1 and Sck2 [33]. A seemingly universal function of TORC1 is to regulate protein synthesis in response to nutrients and stresses [10] and this function is achieved in part by phosphorylation and activation of the S6 kinases, which phosphorylate substrates such as the ribosomal protein S6 [32]. Psk1, Sck1 and Sck2 all phosphorylate the *S*. *pombe* S6 proteins Rps601 and Rps602 [12, 33].

We reasoned that if ComboDT enhances lifespan by down-regulating TORC1 activity, then the CLS of *psk1Δ* cells should be lower than WT cells during drug treatment. Our data support this hypothesis because the CLS of ComboDT-*psk1Δ* cells is reduced compared to ComboDT-WT cells (Fig. 2B). However, CLS in ComboDT- *psk1Δ* cells is not reduced down to the level seen in untreated WT or *psk1Δ* cells, which have identical lifespans (Fig. 2B), indicating that the combination treatment is modulating other TORC1-dependent outputs or other pathways independent of TORC1 to produce maximal effects on lifespan. Likewise, treatment with either myriocin or rapamycin does increase the CLS of *psk1Δ* cells, but only to the level seen in WT cells, indicating that ComboDT affects *psk1Δ* cells in ways that the individual drugs cannot do.

Examination of stress resistance in ComboDT-treated *psk1Δ* cells shows that they are less resistant to heat and oxidative stress than ComboDT-treated WT cells (Fig. 2C). Since drug treatment increases heat and oxidative stress in the absence of Psk1 function, the drugs must be modulating additional pathways to promote stress resistance and data shown below add additional support to this hypothesis. Lastly, we examined the effect of ComboDT on phosphorylation of Myc-tagged Psk1 and the ribosomal S6 proteins Rps601/602 [Rsp6s [33]]. Myriocin by itself does not change the mobility of Psk1-13Myc nor does it change the phosphorylation level of Rps6s (Rsp6-P) (Fig. 2D, compare lanes 1 and 2), indicating that the drug has no measurable effect on the TORC1 pathway. In contrast, low dose rapamycin treatment causes Psk1-13Myc to migrate faster, representing dephosphorylation, and it eliminates the Rps6-P signal (Fig. 2D, lane 3), as seen previously with short term, acute rapamycin treatment [33, 40]. The same results are seen with ComboDT (Fig. 2D, lane 4) and this is probably due to rapamycin, not the drug combination. From the data shown in Fig. 2, we conclude that ComboDT down-regulates the TORC1-Psk1/S6K pathway in *S*. *pombe* and that this reduction plays a role in extending lifespan. But our data also show that the drug combination must be influencing other substrates of this pathway and perhaps other signaling pathways because the *tco89Δ* and *psk1Δ* mutations do not entirely eliminate the effect of ComboDT on lifespan.

**Fig 3:**
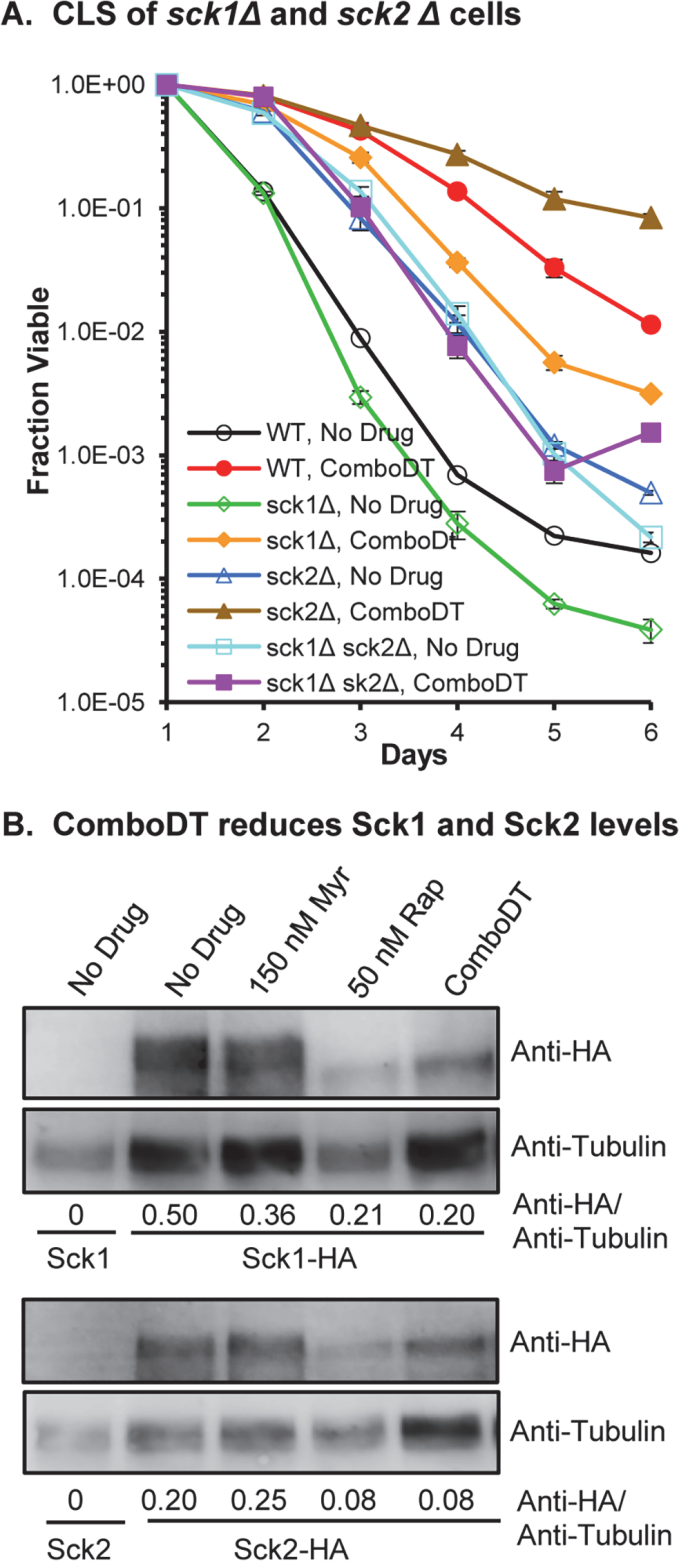
Both Sck1 and Sck2 are required for lifespan enhancement by ComboDT. (A) The effect of drugs on CLS are shown for WT, *sck1Δ*, *sck2Δ* and *sck1Δ sck2Δ* double mutant cells. (B) The effect of drugs on TORC1-regulated phosphorylation of HA-tagged Sck1 and Sck2 was examined by immunoblotting. Numbers below each lane indicate the ratio of the Anti-HA signal to the Anti-Tubulin signal, which served as a protein loading control. This experiment was performed twice with duplicate cultures and similar results were observed each time.

The Sck1 and Sck2 protein kinases were also examined since they are substrates of TORC1 with roles in lifespan [21, 22, 33]. We find that the CLS of untreated *sck1Δ* cells is lower than WT cells, but they do respond to ComboDT and live longer than untreated mutant cells, although not as long as ComboDT-WT cells (Fig. 3A). Untreated *sck2Δ* cells live longer than WT cells, similar to what others have observed [21, 22, 24]. They too respond to ComboDT and have an enhanced CLS which, surprisingly, is higher than ComboDT-WT cells (Fig. 3A). Finally, the CLS of untreated *sck1Δ sck2Δ* double mutant cells is like the *sck2Δ* cells, but the CLS of the double mutant is not enhanced by ComboDT. These data point to Sck1 and Sck2 as vital components in the mechanisms of lifespan enhancement controlled by ComboDT, with each kinase providing unique inputs.

To show that ComboDT actually affects the Sck1 and Sck2 proteins, we examined these kinases by immunoblotting as done previously [33]. We find that myriocin treatment causes a decrease in the HA signal of HA-tagged Sck1 compared to no drug treatment, but does not affect HA-Sck2. Data were quantified by measuring the ratio of the HA signal to tubulin, an internal control protein (Fig. 3B). On the other hand, rapamycin by itself decreases the HA signal of both Sck1 and Sck2 and does so to about the same extent as does ComboDT, indicating that the rapamycin component of the ComboDT is primarily responsible for the reduction of Sck2. For Sck1 rapamycin may be solely responsible for the reduction in protein level, but myriocin alone does have an effect and may contribute to the reduction seen with ComboDT (Fig. 3B). These data suggest that ComboDT modulates CLS in part by reducing the level of the Sck1 and Sck2 proteins to a level that allows the drugs to produce a synergistic increase in lifespan.

### ComboDT Requires the PKA Pathway to Maximally Enhance Lifespan

**Fig 4:**
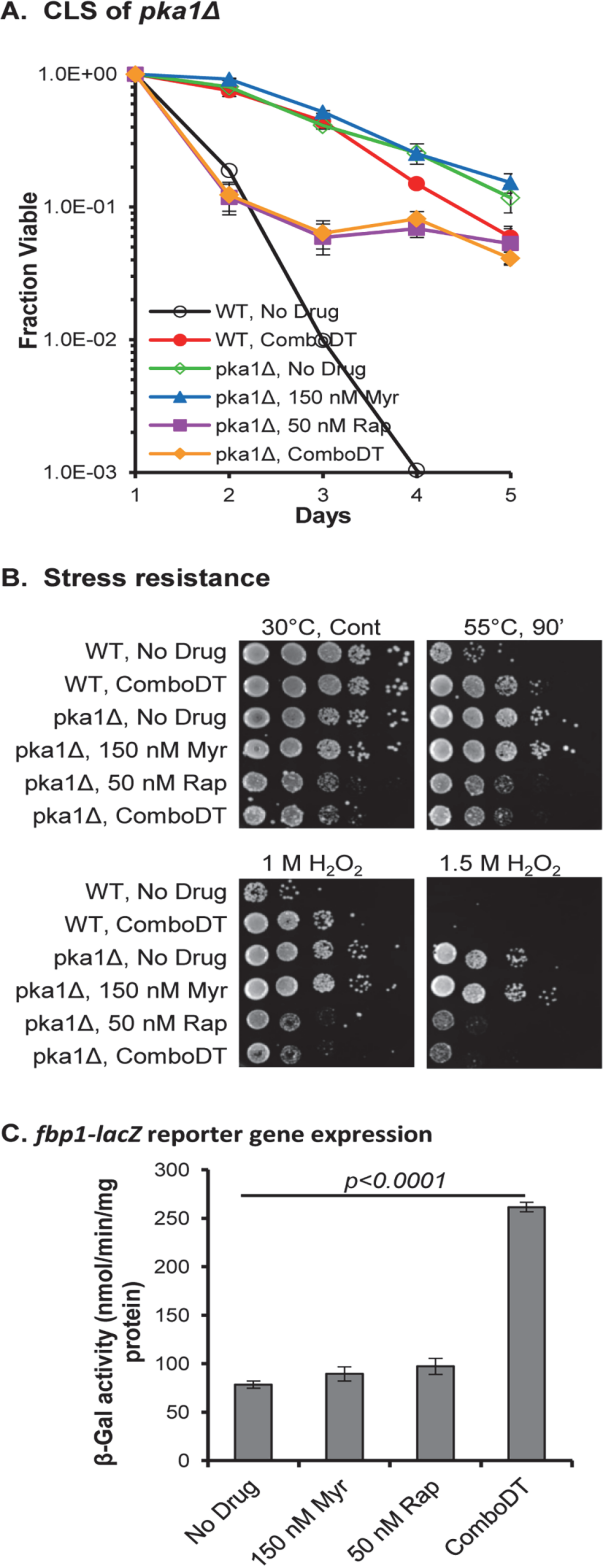
The PKA pathway is vital for ComboDT to enhance CLS and stress resistance. (A) The effect of drugs on the CLS of WT and *pka1Δ* cells are shown. (B) Spot-dilution (10-fold from left to right) assays show the response of WT and *pka1Δ* cells from CLS day 1 to heat or hydrogen peroxide stress treatment. (C) Influence of drugs on expression of the glucose-repressible *fbp1-lacZ* reporter gene are shown.

Down-regulation of the glucose-responsive PKA pathway in *S*. *pombe* [21] as well as in *S*. *cerevisiae* [42, 43] and mice [44] enhances lifespan. Generally, a high level of glucose activates the PKA pathway and suppresses longevity (promotes aging) while a low level of glucose, as in some dietary restriction protocols including in fission yeasts [23], promote longevity in ways that are only partially understood, even in budding yeasts where this pathway has been examined in greatest detail [45–47]. To determine if the PKA pathway in *S*. *pombe* is used by ComboDT to enhance CLS, we examined a *pka1Δ* mutant and matched WT strain that have been used in previous lifespan studies [21]. As shown before, the CLS of untreated *pka1Δ* cells is greatly enhanced relative to untreated WT cells but only slightly enhanced compared to ComboDT-WT cells (Fig. 4A). ComboDT reduces the CLS of *pka1Δ* cells about 10-fold (Fig. 4A) and this is likely due to rapamycin in ComboDT, because rapamycin treatment reduces the CLS of *pka1Δ* cells to about the same extent as ComboDT. Myriocin treatment has no effect on the CLS of *pka1Δ*. In addition, the resistance of *pka1Δ* cells to heat and oxidative stress is reduced by rapamycin treatment or ComboDT while myriocin treatment slightly increases stress resistance (Fig. 4B). From these data we conclude that the PKA pathway is necessary for ComboDT to produce its maximal effect on lifespan and heat and oxidative stress resistance. These data also reveal that a low dose of either myriocin or rapamycin modulate lifespan and stress resistance in distinct ways.

As another way to evaluate whether ComboDT modulates the PKA pathway, we examined expression of the *fbp1* gene, encoding fructose 1,6-bisphosphatase, a glucose-repressible enzyme in the gluconeogenic pathway [48]. A strain carrying an *fbp1-lacZ* reporter gene was used for these assays and β-galactosidase activity was measured in cells grown in medium with 2% glucose to repress gene expression. Neither low dose rapamycin nor myriocin derepress *fbp1-lacZ* expression above the basal level seen in untreated, glucose-repressed cells (Fig. 4C). In contrast, ComboDT produces a statistically significant increase in *fbp1-lacZ* expression, showing that ComboDT down-regulates the PKA pathway even during glucose repression.

Low concentrations of glucose promote down-regulation of the PKA pathway and high expression of the *fbp1-lacZ* gene [48]. To determine how effective ComboDT is in down-regulating the PKA pathway compared to low glucose levels, we measured *fbp1-lacZ* expression in cells gown in medium containing 0.1% glucose and 3% glycerol to fully derepress gene expression. Under these conditions, *fbp1-lacZ* expression is nearly 100-fold higher (data not shown) than the value shown in Fig. 4C for untreated cells and 25-fold higher than for ComboDT cells, both cultured with 2% glucose. Thus, ComboDT only partially down-regulates the PKA pathway compared to the fully derepressed conditions promoted by low glucose levels, but the partial decrease in PKA activity is enough to foster lifespan extension.

Because the PKA pathway is influenced by glucose, it is possible that ComboDT enhances CLS by slowing the rate of glucose utilization or by reducing pathway activity in some other manner. We examined the effect of ComboDT on the level of glucose in culture medium at 0, 24 and 48 hrs of incubation for WT, *pka1Δ* and *sty1Δ* cells. In all strains the concentration of glucose at 48 hrs was very low and was not statistically different in drug-treated or untreated cells (S4 Fig.). The residual concentration of glucose in the cultures containing *pka1Δ* cells was about 2-fold higher than in the other strains, but even this concentration is very low and unlikely to explain the influences of ComboDT on *pka1Δ* cells. Glucose utilization in untreated *pka1Δ* and *sty1Δ* cells is slightly less than in WT cells at the 24 hr time point and in all strains the effect of ComboDT is to slightly reduce glucose utilization. But these relatively small differences are erased by 48 hrs of growth and it seems unlikely that ComboDT enhances lifespan primarily by modulating glucose utilization. Our data do not, however, rule out the possibility that effects of ComboDT on glucose utilization or signaling contribute to enhanced lifespan.

### Roles for Sty1 in Lifespan Enhancement by ComboDT

**Fig 5:**
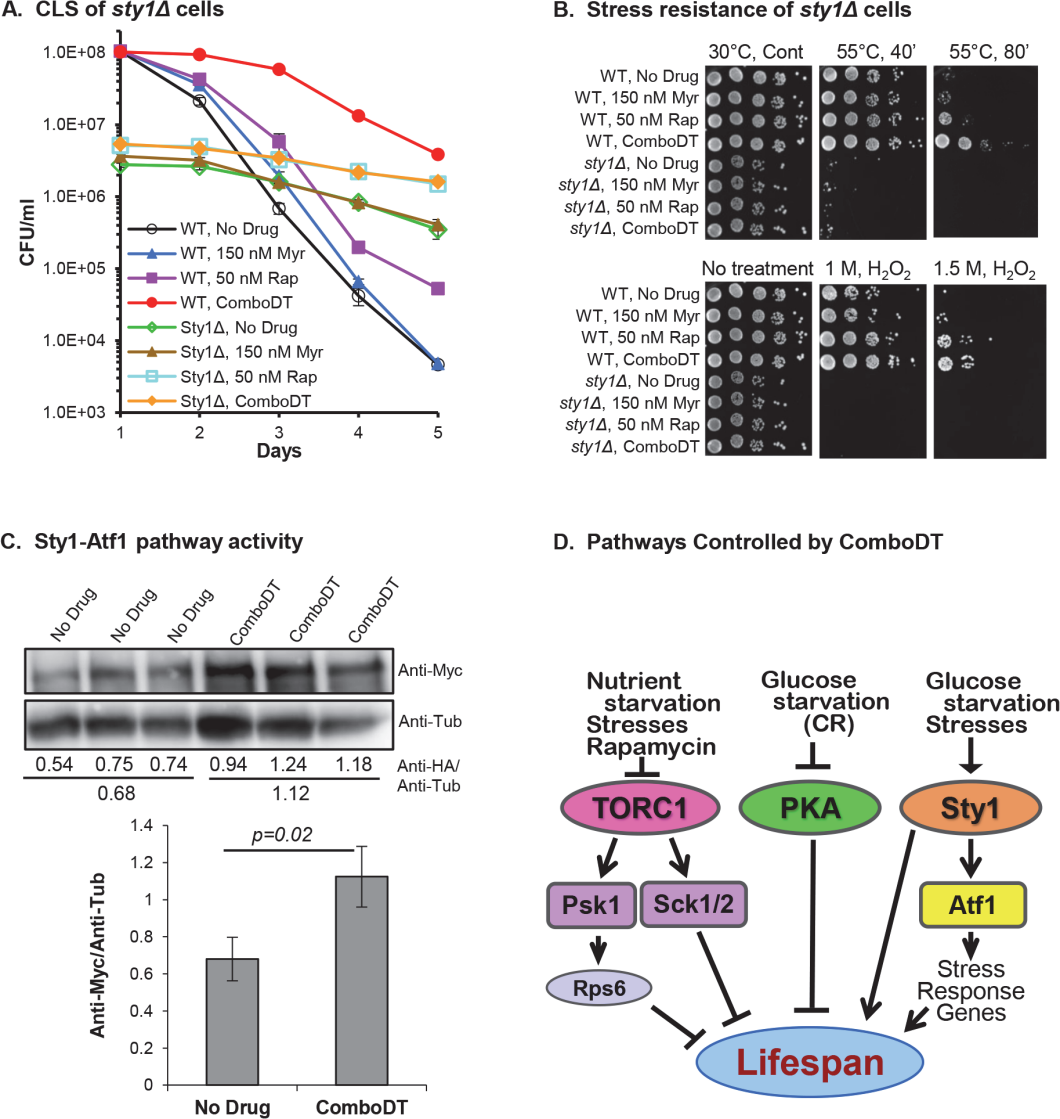
Effects of ComboDT on the Sty1 pathway. (A) The fraction of viable *sty1Δ* cells at CLS day 1 is lower than in WT cells but ComboDT enhances viability in both types of cells. (B) Sty1 function is required for stress resistance in untreated or drug-treated *sty1Δ* cells. (C) Effect of ComboDT on Atf1 protein abundance in cells expressing Myc-tagged Atf1. Upper two panels are immunoblot data for Atf1 (Anti-Myc) and the tubulin (Anti-Tub) loading control. Below each lane is the pixel density of the Anti-Myc value divided by the value for Anti-Tub. The average of the triplicate samples is shown below the individual values. Average values are plotted in the bottom panel along with statistical significance. (D) Diagram of the signaling pathways identified in this study that are modulated by ComboDT to increase lifespan in WT fission yeast cells. Controlling factors are listed above each pathway with ones that reduce pathway activity indicated by blunt-ended lines and ones that increase pathway activity indicated by arrowheads. Psk1, Sck1 and Sck2 are the fission yeast orthologs of mammalian S6 kinases and Sty1 is the ortholog of mammalian p38 MAP kinase involved in inflammatory and other stress responses. Ways in which myriocin might affect these pathways are presented in the Discussion.

The Sty1/Spc1 MAP kinase in fission yeasts is essential for protection against a wide range of environmental stresses [49, 50]. The protein is also necessary for CR, imposed by a low concentration of glucose, to increase CLS [24]. Using the same *sty1Δ* and WT strains as were used to study the effect of CR on lifespan [24], we find that *sty1Δ* cells treated or not treated with drugs grow to about the same A600_nm_ value as WT cells (S2 Fig.), however, their viability at CLS day 1 (48 hrs incubation) is reduced to about 40% below the WT value (Fig. 5A and 5B). To account for this phenotype, noted previously in mutants defective in the Wis1-Sty1-Atf1 pathway [51, 52], and to better reveal the effect of drugs on viability, CLS data are plotted as colony forming units/ml rather than as a fraction of the CLS day 1 value.

Viability in untreated *sty1Δ* cells falls less than 10-fold over the 5 day time frame of the assay, showing that the mutant cells that manage to remain viable during entry into stationary phase are in a metabolic state that maintains viability in stationary phase far better than occurs in untreated WT cells, whose viability falls 10,000-fold over the 5 day assay period (Fig. 5A). Rapamycin and ComboDT each enhance the viability of *sty1Δ* cells at CLS day 1 along with lowering the loss of viability to about a 3-fold decrease (compared to ∼10-fold without drug treatment) over the course of the assay whereas myriocin treatment has no effect on the viability of *sty1Δ* cells.

In contrast to the enhancing effect of rapamycin and ComboDT on the CLS of *sty1Δ* cells, the drugs have little or no enhancing effect on the exceptionally low resistance of *sty1Δ* cells to heat or oxidative stress (Fig. 5B), showing that Sty1 is required for resistance to such stresses, as observed previously [49, 51–54]. Similar to the data shown in Fig. 5A, the spot dilution assays shown in the left panels of Fig. 5B (30°C, Cont) reveal that there are fewer viable *sty1Δ* cells than WT cells upon entry into stationary phase.

Activation of the Sty1 pathway by environmental stresses prompts Sty1 to transpose from the cytosol to the nucleus where it phosphorylates and activates the Atf1 transcription factor [49, 51, 55–57]. Activation of Atf1 can be monitored by immunoblotting of Myc-tagged Atf1 [55, 57]. If ComboDT is activating the Sty1-Atf1 pathway, then the level of Atf1–12Myc protein should be higher in drug-treated than in untreated cells. We find that ComboDT does produce a statistically significant increase in Atf1-12Myc protein, indicating that ComboDT is activating the Sty1-Atf1 pathway. These results are consistent with previous data showing that the level of Atf1 increases during oxidative and osmotic stress [58], and are also consistent with ComboDT inducing expression of the *fbp1-lacZ* reporter gene (Fig. 4C), since Atf1 promotes *fbp1* ^*+*^ expression [51]. In summary, the data presented in Fig. 5 show that Sty1 is necessary to promote survival during entry into stationary phase based upon the nearly 40% loss of survival in *sty1Δ* cells. ComboDT can boost survival 2 to 3-fold in *sty1Δ* cells, although it is still below the level of WT cells (Fig. 5A, Day 1). ComboDT also increases the survival of *sty1Δ* cells over the 5 day time span of the CLS assay, thus, the drugs are not entirely dependent on the Sty1 pathway to enhance survival, but the pathway is necessary for ComboDT to produce a synergistic effect on survival.

## Discussion

The data presented here demonstrate that a low dose combination of rapamycin and myriocin produces a synergistic increase in the CLS of S. *pombe* cells (Fig. 1A). These results along with our published data showing that the same drug combination promotes a synergistic increase in the CLS of *S*. *cerevisiae* cells, support the idea that ComboDT extends lifespan by modulating evolutionarily conserved mechanisms, since these two yeasts diverged about 330–420 million years (Myr) ago by one estimate [59] and by 1,144 Myr years ago by another estimate [60]. Furthermore, some features of fission yeasts relevant to aging and longevity including a strict requirement for aerobic growth, lack of a diauxic shift and a glyoxylate cycle and the presence of the TORC1 regulatory proteins Tsc1/2 and the Rheb GTPase are more like mammals than budding yeasts [61]. Likewise, gene regulation through chromatin modification and RNA interference in fission yeast are more related to metazoans than to budding yeasts [62]. Finally, a recent study examined the degree to which protein-protein interactions for orthologous proteins are conserved between *S*. *cerevisiae* and *S*. *pombe* [63]. This work is especially significant to aging and longevity because of the focus on proteins involved in stress responses and signal transduction. The results showed that protein interactions tend not to be conserved in these two species, rather they are rewired to form new interactions, which are believed to drive divergence. Such differences between budding and fission yeasts suggest that ComboDT is able to modulate not only conserved signaling pathways, but can also exploit species-specific pathway rewiring and consequent novel attributes to extend lifespan. Thus, it is not unreasonable to expect that ComboDT can slow aging and perhaps enhance lifespan in multicellular eukaryotes.

We identified conserved signaling pathways used by ComboDT to enhance lifespan. Because the TORC1 pathway controls aging and longevity in many organisms [1, 3, 64] and is used by ComboDT to enhance CLS in *S*. *cerevisiae*, we hypothesized that ComboDT uses TORC1 to control fission yeast lifespan. Our data support the hypothesis (Fig. 2A and 2D). TORC1 phosphorylates many substrates but the S6 kinases are the ones with the best documented roles in longevity [1, 3, 41]. Examination of the three fission yeast orthologs of mammalian S6 kinases, Psk1, Sck1 and Sck2, [21, 22], shows that they are required for ComboDT to enhance lifespan (Figs. 2B and 3A).

We find that Psk1 is not needed for a normal lifespan, the first such demonstration, but is needed for ComboDT to produce its maximal effect on CLS (Fig. 2B, yellow curve). Intriguingly, our results suggest that Psk1 is not necessary to produce synergy (Fig. 2B, yellow curve), however, a more thorough analysis will be required to prove this possibility. Immunoblotting of Psk1-13Myc and one of its substrates, the ribosomal S6 protein, serve to verify that ComboDT does indeed down-regulate the Psk1 arm of the TORC1 pathway. Furthermore, this effect is caused by the rapamycin component of ComboDT (Fig. 2D). Previously a 1 hr exposure (less than one cell division) of cells to 50 nM rapamycin did not cause a shift in the mobility of Psk1-13Myc [33] whereas the same drug concentration causes a complete shift in our assays (Fig. 2D). This difference is likely to result from the longer drug-exposure time (4–5 cell divisions) used in our assays. It has been suggested previously that Psk1, Sck1 and Sck2 are S6-type kinases that have partially overlapping functions (Nakashima et al. 2012). Such redundancies may explain the normal lifespan of *psk1Δ* cells that we find and the apparent lack of a role in producing synergy.

Data for the Sck1 and Sck2 kinases are informative. ComboDT increases the CLS of *sck1Δ* cells almost to the degree seen in WT cells and, surprisingly, the CLS of drug-treated *sck2Δ* cells is greater than WT cells (Fig. 3A). In contrast, ComboDT does not increase CLS in *sck1Δ sck2Δ* double mutant cells, demonstrating that both the Sck1 and Sck2 arms of TORC1 signaling are essential for ComboDT to enhance lifespan. Immunoblot analysis of Sck protein level verified that ComboDT does reduce TORC1 activity. As with Psk1, it is the rapamycin component of ComboDT that controls the concentration of Sck1 and Sck2, although the level of Sck1 is reduced by myriocin to about half the value bound with rapamycin (Fig. 3B).

The CLS of *sck1Δ* cells was found previously to be similar to WT in culture media contain 2–3% glucose [21] whereas we see a slight reduction in CLS (Fig. 3A) that may be due to small differences in culture conditions. The enhanced CLS of *sck2Δ* and *sck1Δ sck2Δ* cells that we observe in untreated cells (Fig. 3A) has been seen previously [21, 22, 65]. The CLS of *sck2Δ* cells was shown to be enhanced by deleting *pka1* [21] and we observe a similar effect when *sck2Δ* cells are treated with ComboDT (Fig. 3A), which, based on data shown in Fig. 4C, is likely due to the drug combination down-regulating the Pka1 pathway. In medium with 5% glucose, *sck1Δ* and *sck2Δ* cells have an enhanced lifespan [22], similar to what we see with ComboDT and further study is needed to determine if the underlying mechanisms for lifespan enhancement are similar.

The Pka1 pathway, along with the Sck2 pathway, were the first in fission yeasts to be shown to regulate CLS [21] and our data implicate both pathways as vital elements in the mechanisms used by ComboDT to enhance lifespan. Similar to previous results, the CLS of untreated *pka1Δ* cells is greater than untreated WT cells (Fig. 4A). In fact, untreated *pka1Δ* cells survive slightly longer than ComboDT-treated WT cells (Fig. 4A). Rapamycin alone or in ComboDT causes a reduction in the CLS of *pka1Δ* cells, although cells still live significantly longer than untreated cells (Fig. 4A), indicating that long CLS of *pka1Δ* cells depends on the TORC1 pathway. Similar to the CLS data, rapamycin and ComboDT also reduce the heat and oxidative stress resistance of *pka1Δ* cells (Fig. 4B). It has between suggested that Pka1 and Sck2, operating downstream of TORC1, function in parallel pathways that control lifespan [21, 23] and our data are consistent with such a model, particularly with data showing negative genetic interactions between mutants in the two pathways [40].

Our data demonstrate that the Sty1 MAP kinase is necessary for ComboDT to modulate growth and metabolism so that most cells remain viable upon entry into stationary phase and continue to remain viable longer than untreated *sty1Δ* or WT cells (Fig. 5A). These data are akin to the role of Sty1 in lifespan extension induced by CR [24] and they, along with our results for the PKA and TORC1 pathways, show that ComboDT uses several of the same pathways to enhance lifespan that CR uses.

Resistance to environmental stresses is often associated with longevity, but the correlation does not always hold true in model organisms [66, 67]. A lack of correlation between longevity and oxidative stress resistance in fission yeasts is supported by studies of long-lived strains [40] and also by our studies with ComboDT. While WT fission yeasts treated with ComboDT are more resistant to heat and oxidative stress (Fig. 1B), these phenotypes may not be essential for increased CLS. For example, heat or hydrogen peroxide treatment strongly reduce the viability of *pka1Δ* and *sty1Δ* cells yet CLS is enhanced by ComboDT in these mutants to a level that far exceeds the CLS of untreated WT cells (Figs. 4A and 5A). Understanding the mechanisms that enable ComboDT to enhance lifespan in *pka1Δ* and *sty1Δ* cells may provide key insights into novel ways to moderate aging and promote longevity despite low stress resistance.

Lifespan enhancement by calorie restriction or growth in a non-repressing carbon source like glycerol [21] produce a large increase in CLS similar to what we find with ComboDT and the underlying mechanisms may have similarities. For example, both low glucose (0.2%) [23, 48] and ComboDT (Fig. 4C) induce expression of the *fbp1-lacZ* reporter gene, indicative of down-regulation of the Pka1 pathway. Likewise, both treatments increase protection against oxidative stress produced by hydrogen peroxide treatment [23] (Fig. 1B). More similarities between the mechanisms that increase lifespan during CR or growth in a non-repressing carbon source and ComboDT are likely to exist. Nitrogen starvation is an established way to promote quiescence or survival during stationary phase [61]. Although we did not specifically determine if nitrogen starvation and ComboDT work in similar ways to promote lifespan, it seems likely that they have some features in common since both require functions of Sty1.

Results from these studies are summarized in Fig. 5D. TORC1, PKA and Sty1 are the three pathways we have identified as being required for ComboDT to produce a synergistic increase in CLS in WT cells. These signal transduction pathways respond to glucose other nutritional starvation conditions along with environmental stresses such as reactive oxygen species and high temperature, indicating that ComboDT works in part by mimicking the effects of these types of stresses. These low level stress effects are similar to what ComboDT does to *S*. *cerevisiae* cells, indicating that the drugs promote mild, sub-lethal stress or hormesis as at least part of the mechanism for increasing lifespan, similar to what is believed to occur in other longevity promoting strategies including rapamycin treatment and dietary or calorie restriction. [68–70].

Future studies will be required to identify other signaling pathways and cellular processes regulated by ComboDT that promote longevity. Many complex cellular processes including cell growth and size control, protein synthesis, autophagy and other processes dependent on membrane trafficking, along with proteostasis, nitrogen, carbon and energy metabolism, and stress resistance are likely to respond to ComboDT. A recent systematic screen for mutants resistant to TORC1 inhibition by rapamycin plus caffeine gives insight into the multifaceted manner in which this pathway and its downstream target Sck2 respond to a drug combination that enhances lifespan [40]. The combination of rapamycin and myriocin may produce more complex outputs to enhance lifespan because myriocin works by lowering the rate of sphingolipid synthesis and, when combined with effects of reduced TORC1 activity on other lipid biosynthesis pathways, has the potential to alter lipid signaling molecules, membrane trafficking, membrane protein activity and phase transitions in membranes thereby perturbing multiple cellular compartments [18, 71]. We speculate that such changes along with alterations in metabolism coalesce to enhance lifespan.

## Material and Methods

### Strains and media

*S*. *pombe* strains used in this study are listed in S1 Table. Yeast extract complete medium (YES) contained yeast extract (5 g/L, BD, Difco), supplemented with 222 mg/L of adenine (A), uracil (U), leucine (L) and histidine (H), and glucose (2%)[23].

### CLS assay

CLS was assayed as described previously [23]. Cells grown overnight at 30°C to saturation in YES medium, were diluted into 25 ml of medium (125 ml flask) to give an initial A_600nm_ of 0.005. Cultures were incubated at 30°C in an air bath shaker (220 rpm) for two days (48 hrs) and cell viability was measured (CLS day 1) by diluting and spreading cells on YES plates. Colonies were counted after 2–3 days of incubation at 30°C (4–5 days of incubation for slow growing *pka1Δ* cells) and expressed as a fraction of the day 1 value. Statistical significance was determined by using the two-tailed Student’s *t*-test. For drug treatment, cells were cultured in medium containing a final concentration of 0.29% (v/v) ethanol with or without drug(s). This was done by first adding a calculated volume of 95% ethanol to the medium followed by diluting a stock solution of drug (50 μg Myriocin/ml or 25 μg Rapamycin/ml, both in 95% EtOH) to give a concentration of 150 nM Myriocin (Sigma) or 50 nM Rapamycin (LC Laboratories) or both.

### Stress resistance assay

Cells were grown as described for a CLS assay in YES medium for the time indicated in Figure legends and then diluted to give an A600nm of 1.0 units/ml followed by treatment with hydrogen peroxide for 60 min at room temperature with mixing. Serially diluted (10-fold) cells were spotted (4 μl) onto YES plates and incubated at 30°C for 2–3 days. For heat stress resistance, cells were serially diluted (10-fold dilution starting with an A600nm = 1), spotted onto YES plates, and incubated at either 55°C (heat-shocked) or 30°C (control) for 60 or 90 min, followed by incubation for 2–3 days at 30°C.

### Protein extraction and western blotting

Cells were grown as described for a CLS assay and cell-free yeast extracts were prepared by using a modified published procedure [58]. Briefly, 20 A_600nm_ units of cells, grown from 0.005 to 1.0 A_600nm_ with or without drug(s), were slowly combined with cold 50% trichloroacetic acid to give a final concentration of 6% followed by incubation on ice for 5 min and then centrifugation for 5 min x 5,000 g. Pellets were washed once with cold water, suspend in 1 ml of 0.2 N NaOH, transferred to a screw-capped microfuge tube and incubated for 5 min at room temperature before centrifugation. Pellets were suspended in sample buffer (0.06 M Tris-HCl, pH 6.8, 5% glycerol, 2% SDS, 1mM EDTA) at a concentration of 10 A_600nm_ units per 100 μl. Samples were heated at 95°C for 8 min, centrifuged and the protein concentration of the supernatant was determined by using the DC protein assay kit (Bio-Rad Laboratories). Samples electrophoresed on SDS-PAGE gel were transferred onto a PVDF membrane (Millipore Immobilon-FL) by using a Bio-Rad semi-dry transfer system. Membrane blocking and antibody binding were done in TBST (20 mM Tris, 150 mM NaCl, 0.1% Tween-20, pH 7.5) containing 5% nonfat dry milk. Primary antibodies were anti-Myc tag (1:2000, Millipore, clone 9E10, Cat.#05–419), anti-phospho-(Ser/Thr) Akt substrate (1:1000, Cell signaling, Cat.#9611), anti-HA tag (1:2000, Sigma, Cat.#H6908) and anti-tubulin (1:2000, Sigma, Cat.#T5168). Second antibodies were anti-Mouse IgG (1:5000, Sigma, Cat.#A3562) or anti-Rabbit IgG (1:5000, Sigma, Cat.#A3687). Fluorescent signals from membranes exposed to an ECF substrate (Amersham Biosciences) were analyzed by using the Bio-Rad ChemiDoc MP Imaging System and quantified by using the Image Lab Software.

### β-Galactosidase assay

β-galactosidase activity was measured in cells grown to log phase (A_600nm_ = 1.0). Cells were collected by centrifugation and suspended in 1 ml of Z buffer (0.06 M Na_2_HPO_4_, 0.04 M NaH_2_PO_4_, 0.01 M KCl, 0.001 M MgSO4, pH 7.0, 0.05 M β-mercaptoethanol (add fresh)). Cells were made permeable by adding 25 μl chloroform and 40 μl 0.1% SDS followed by vortexing. β-galactosidase activity was expressed as nmol/min/mg protein. Statistical analysis was performed by using a two-tailed Student’s *t*-test.

## Supporting Information

S1 Fig Effect of individual drugs on cell growth. WT *S*. *pombe* cells were diluted into culture medium containing the indicated concentration of myriocin (Myr, Panel A) or rapamycin (Rap, Panel B) and grown as described for a CLS assay. Absorbance at 600nm (A600 nm) was measured at the indicated times. Average values for three cultures are show. [100 ng/ml Myr = 250 nM and 10 ng/ml Rap = 10.86 nM].
(PDF)

S2 Fig Effect of ComboDT on cell growth. WT, *pka1Δ* and *sty1Δ* cells were diluted into culture medium +/- ComboDT (50 nM myriocin + 150 nM rapamycin = 60 ng/ml myriocin + 46 ng/ml rapamycin) and grown as described for a CLS assay. Absorbance at 600nm (A600 nm) was measured at the indicated times. Average values for three cultures are show.
(PDF)

S3 Fig Determination of EC50 for individual drugs. (A) EC50 of individual myriocin treatment. Cells were grown as described in a CLS assay for 48 hrs, and survival in a serial dilution series of myriocin (0, 62.25, 124.5, 249, 498, 747, 996, 1494 nM) was measured at a fixed time (time 0, CLS day 1, 100% survival) and again after 32 hrs. The effect of myriocin treatment on survival at 32 hrs was assessed to obtain the median effective concentration (EC50). Nonlinear regression was used to fit the survival versus concentration curve. (B) EC50 for rapamycin treatment. Concentrations of rapamycin are 0, 32.7, 109, 218, 545, 1090, 2180 nM.
(PDF)

S4 Fig Glucose utilization during the first 48 hrs of culture incubation. (A) The concentration of glucose was measured in triplicate cultures of WT, *pka1Δ* and sty1*Δ* cells at the time of inoculation (0 hrs) and after 24 and 48 hrs of incubation at 30°C. Glucose was measured by using a commercial kit (Glucose Oxidase-Catalase Assay, Shanghai Rongsheng Biotech Co. Ltd, China. Cat.: 361500). (B) Average numerical values for the data plotted in A are shown along with statistical evaluation of the indicated comparisons made by using the Students’ t-*test*.
(PDF)

S1 Table Strains used in this study. (DOCX)

S2 Table Statistical analysis of CLS data. (XLSX)
