# Correction: Drug Synergy Drives Conserved Pathways to Increase Fission Yeast Lifespan

**DOI:** 10.1371/journal.pone.0125857

**Published:** 2015-04-29

**Authors:** Xinhe Huang, Markos Leggas, Robert C. Dickson

The email address for author Xinhe Huang is incorrect. The correct email address is: xinhehuang@home.swjtu.edu.cn.

The following information is missing from the Funding section: This study was supported by the Fundamental Research Funds for the Central Universities of China grant 2682014RC14 to XH.
