# Supplementary material for: Drug Synergy Drives Conserved Pathways to Increase Fission Yeast Lifespan
Source: PLoS One. 2015 Mar 18;10(3):e0121877. doi: 10.1371/journal.pone.0121877 (PMC4364780; doi:10.1371/journal.pone.0121877)
Supplement: S1 Fig — WT S. pombe cells were diluted into culture medium containing the indicated concentration of myriocin (Myr, Panel A) or rapamycin (Rap, Panel B) and grown as described for a CLS assay. Absorbance at 600nm (A600 nm) was measured at the indicated times. Average values for three cultures are show. [100 ng/ml Myr = 250 nM and 10 ng/ml Rap = 10.86 nM]. (PDF) [file pone.0121877.s001.pdf]

**S1 Fig.**

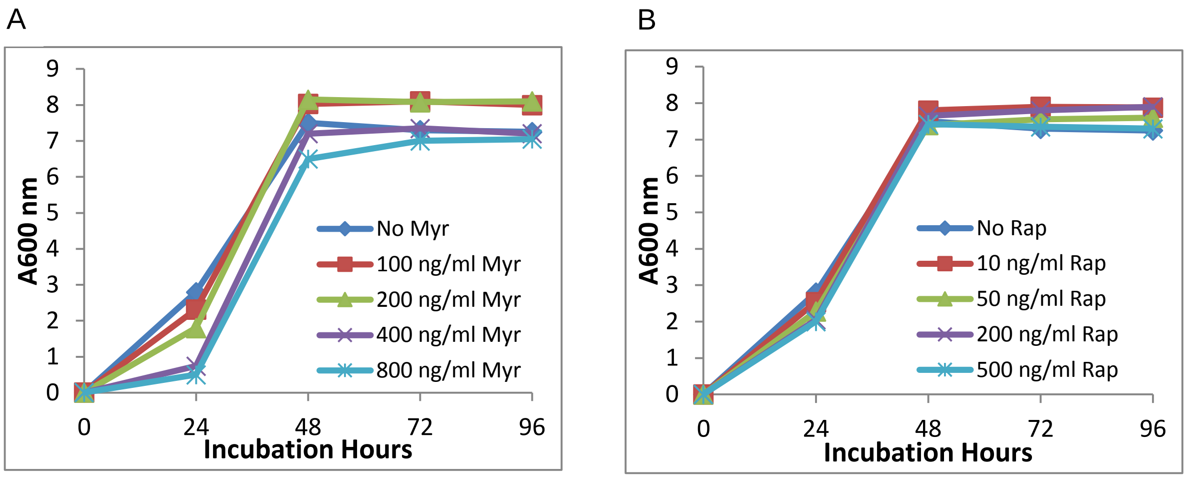


**S1 Fig. Effect of individual drugs on cell growth.** WT *S. pombe* cells were diluted into culture medium containing the indicated concentration of myriocin (Myr, Panel A) or rapamycin (Rap, Panel B) and grown as described for a CLS assay. Absorbance at 600nm (A600 nm) was measured at the indicated times. Average values for three cultures are show. [100 ng/ml Myr = 250 nM and 10 ng/ml Rap = 9.6 nM].
