# Supplementary material for: Drug Synergy Drives Conserved Pathways to Increase Fission Yeast Lifespan
Source: PLoS One. 2015 Mar 18;10(3):e0121877. doi: 10.1371/journal.pone.0121877 (PMC4364780; doi:10.1371/journal.pone.0121877)
Supplement: S2 Fig — WT, pka1Δ and sty1Δ cells were diluted into culture medium +/- ComboDT (50 nM myriocin + 150 nM rapamycin = 60 ng/ml myriocin + 46 ng/ml rapamycin) and grown as described for a CLS assay. Absorbance at 600nm (A600 nm) was measured at the indicated times. Average values for three cultures are show. (PDF) [file pone.0121877.s002.pdf]

S2 Fig.

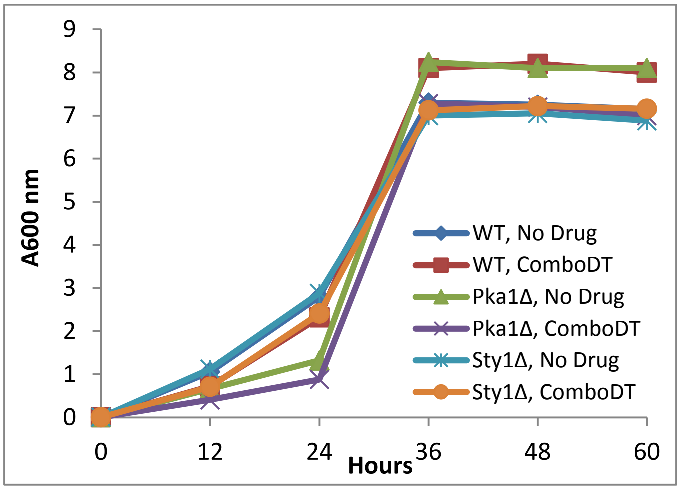

S2 Fig. Effect of ComboDT on cell growth. WT, *pka1Δ* and *sty1Δ* cells were diluted into culture medium +/- ComboDT (50 nM myriocin + 150 nM rapamycin = 60 ng/ml myriocin + 46 ng/ml rapamycin) and grown as described for a CLS assay. Absorbance at 600nm (A600 nm) was measured at the indicated times. Average values for three cultures are show.
