# Supplementary material for: Drug Synergy Drives Conserved Pathways to Increase Fission Yeast Lifespan
Source: PLoS One. 2015 Mar 18;10(3):e0121877. doi: 10.1371/journal.pone.0121877 (PMC4364780; doi:10.1371/journal.pone.0121877)
Supplement: S3 Fig — (A) EC50 of individual myriocin treatment. Cells were grown as described in a CLS assay for 48 hrs, and survival in a serial dilution series of myriocin (0, 62.25, 124.5, 249, 498, 747, 996, 1494 nM) was measured at a fixed time (time 0, CLS day 1, 100% survival) and again after 32 hrs. The effect of myriocin treatment on survival at 32 hrs was assessed to obtain the median effective concentration (EC50). Nonlinear regression was used to fit the survival versus concentration curve. (B) EC50 for rapamycin treatment. Concentrations of rapamycin are 0, 32.7, 109, 218, 545, 1090, 2180 nM. (PDF) [file pone.0121877.s003.pdf]

S3 Fig.

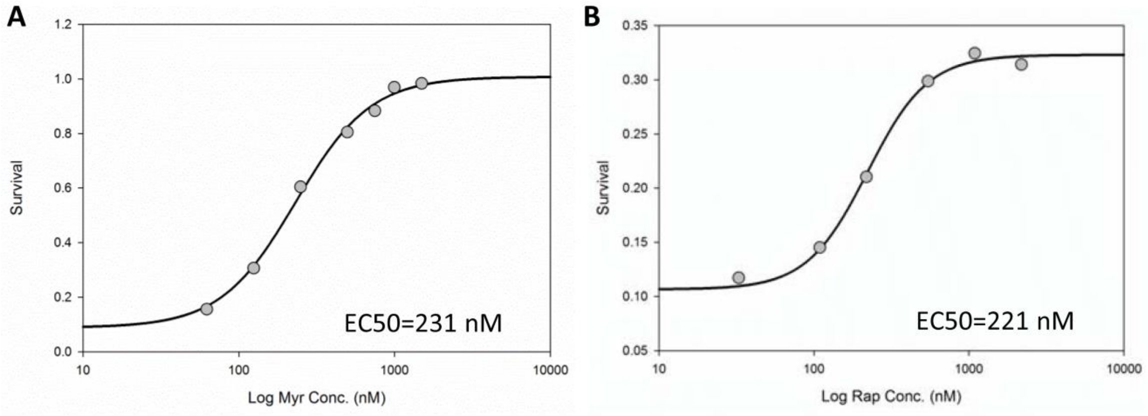

S3 Fig. Determination of EC50 for individual drugs. (A) EC50 of individual myriocin treatment. Cells were grown as described in a CLS assay for 48 hrs in different myriocin concentrations (0, 62.25, 124.5, 249, 498, 747, 996, 1494 nM). Survival was measured at time 0 (CLS day 1, 100% survival) and again after 32 hrs of incubation. The effect of myriocin treatment on survival at 32 hrs was assessed to obtain the median effective concentration (EC50). Nonlinear regression was used to fit the survival versus concentration curve. (B) EC50 for rapamycin treatment. Concentrations of rapamycin are: 0, 32.7, 109, 218, 545, 1090, 2180 nM.
