# Supplementary material for: Drug Synergy Drives Conserved Pathways to Increase Fission Yeast Lifespan
Source: PLoS One. 2015 Mar 18;10(3):e0121877. doi: 10.1371/journal.pone.0121877 (PMC4364780; doi:10.1371/journal.pone.0121877)
Supplement: S4 Fig — (A) The concentration of glucose was measured in triplicate cultures of WT, pka1Δ and sty1Δ cells at the time of inoculation (0 hrs) and after 24 and 48 hrs of incubation at 30°C. Glucose was measured by using a commercial kit (Glucose Oxidase-Catalase Assay, Shanghai Rongsheng Biotech Co. Ltd, China. Cat.: 361500). (B) Average numerical values for the data plotted in A are shown along with statistical evaluation of the indicated comparisons made by using the Students’ t-test. (PDF) [file pone.0121877.s004.pdf]

S4 Fig.

A

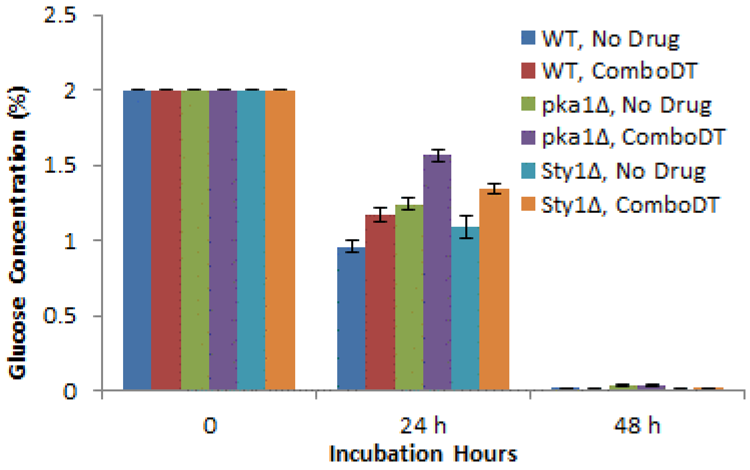

B

Ave		0	24 h	48 h
	WT, No Drug	2	0.963	0.019
	WT, ComboDT	2	1.170	0.015
	pka1Δ, No Drug	2	1.240	0.036
	pka1Δ, ComboDT	2	1.567	0.038
	sty1Δ, No Drug	2	1.090	0.015
	sty1Δ, ComboDT	2	1.343	0.020

		0	24 h	48 h
TTEST	WT, No Drug Vs ComboDT	-	0.005	0.103
	pka1Δ, No Drug Vs ComboDT	-	0.001	0.472
	sty1Δ, No Drug Vs ComboDT	-!	0.013	0.156

S4 Fig. Glucose utilization during the first 48 hrs of culture incubation. (A) The concentration of glucose was measured in triplicate cultures of WT, *pka1Δ* and sty1*Δ* cells at the time of inoculation (0 hrs) and after 24 and 48 hrs of incubation at 30°C. Glucose was measured by using a commercial kit (Glucose Oxidase-Catalase Assay, Shanghai Rongsheng Biotech Co. Ltd, China. Cat.: 361500). (B) Average numerical values for the data plotted in A are shown along with statistical evaluation of the indicated comparisons made by using the Students' t-*test*.
