# Supplementary material for: Drug Synergy Drives Conserved Pathways to Increase Fission Yeast Lifespan
Source: PLoS One. 2015 Mar 18;10(3):e0121877. doi: 10.1371/journal.pone.0121877 (PMC4364780; doi:10.1371/journal.pone.0121877)
Supplement: S1 Table — (DOCX) [file pone.0121877.s005.docx]

Supporting Information

**Table S1**. Strains used in this study.

| **Strains** | **Genotype** | **Source** | **Figure** |
| --- | --- | --- | --- |
| 972 | *h^-^* | [[1](#_ENREF_1)] | 1A, 1B, S1, 2C, 2D, 5A, 5B |
| SP14000 | *h^-^ ade6-M210 ura4-D18 leu1-32* | [[2](#_ENREF_2)] | 2A |
| Tco89Δ | *h^-^ Tco89::kanMX4 ade6-M216ura4-D18leu1-32* | [[3](#_ENREF_3)] | 2A |
| AN0132 | *h^-^ psk1::hphMX* | [[4](#_ENREF_4)] | 2B, 2C, 2D |
| AN0179 | *h^-^ psk1^+^-13myc::hphMX* | [[4](#_ENREF_4)] | 2D |
| KRP14 | *h^-^*  *ade6-M216 ura4-D18 leu1-32 his7-366, sck1Δ::LEU2* | [[5](#_ENREF_5)] | 3A |
| KRP19 | *h^-^*  *ade6-M216 ura4-D18 leu1-32 his7-366, leu1Δ::LEU2* | [[5](#_ENREF_5)] | 3A, 4A, 4B |
| KRP20 | *h^-^*  *ade6-M216 ura4-D18 leu1-32 his7-366, sck2Δ::LEU2* | [[5](#_ENREF_5)] | 3A |
| SP14094 | *h- ade6-M210 leu1-32 ura4-D18 his7-366 sck1::neo^R^ sck2::LEU2* | [[2](#_ENREF_2)] | 3A |
| JUp1204 | *h^90^ FY155* | [[4](#_ENREF_4)] | 3B |
| AN0151 | *h^90^ sck1^+^-3HA-hphMX* | [[4](#_ENREF_4)] | 3B |
| AN0153 | *h^90^ sck2^+^-3HA-hphMX* | [[4](#_ENREF_4)] | 3B |
| CPH453 | *h^-^*  *leu1-32 ura4-D18 his7-366, pka1::ura4^+^* | [[2](#_ENREF_2)] | 4A, 4B |
| FWP70 | *h^+^ ade6-M216 lys1-131 leu1-32 ura4::fbp1-lacZ* | [[6](#_ENREF_6)] | 4C |
| AV18 | *h^-^ sty1::kanMX* | *[*[*1*](#_ENREF_1)*]* | 5A, 5B |
| FG2156 | *h^−^ atf1-12 myc (ura4^+^)* | [[7](#_ENREF_7)] | 5C |

**References:**

1. Zuin A, Carmona M, Morales-Ivorra I, Gabrielli N, Vivancos AP, et al. (2010) Lifespan extension by calorie restriction relies on the Sty1 MAP kinase stress pathway. EBMO J 29: 981-991.

2. Roux AE, Quissac A, Chartrand P, Ferbeyre G, Rokeach LA (2006) Regulation of chronological aging in *Schizosaccharomyces pombe* by the protein kinases Pka1 and Sck2. Aging Cell 5: 345-357.

3. Rallis C, Codlin S, Bahler J (2013) TORC1 signaling inhibition by rapamycin and caffeine affect lifespan, global gene expression, and cell proliferation of fission yeast. Aging Cell 13: 563–573.

4. Nakashima A, Otsubo Y, Yamashita A, Sato T, Yamamoto M, et al. (2012) Psk1, an AGC kinase family member in fission yeast, is directly phosphorylated and controlled by TORC1 and functions as S6 kinase. J Cell Sci 125: 5840-5849.

5. Chen BR, Runge KW (2009) A new *Schizosaccharomyces pombe* chronological lifespan assay reveals that caloric restriction promotes efficient cell cycle exit and extends longevity. Exp Gerontol 44: 493-502.

6. Hoffman CS, Winston F (1990) Isolation and characterization of mutants constitutive for expression of the fbp1 gene of *Schizosaccharomyces pombe*. Genetics 124: 807-816.

7. Gaits F, Degols G, Shiozaki K, Russell P (1998) Phosphorylation and association with the transcription factor Atf1 regulate localization of Spc1/Sty1 stress-activated kinase in fission yeast. Genes Dev 12: 1464-1473.
